# Clinical Notes on Characteristics

**Published:** 1888-05

**Authors:** C. Carleton Smith, Edmund J. Lee

**Affiliations:** Philadelphia; Philadelphia


### CLINICAL NOTES ON CHARACTERISTICS.

C. Carleton Smith, M. D., } Philadelphia.

Edmund J. Lee, M. D., }

### AILANTHUS.

Mental.—Indifference; confusion of mental faculties, reading and counting are difficult. Anxiety, irritability, depression of spirits. In low forms of fever, stupor, delirium, and restlessness. Following a suppression of the scarlatina rash there were stupor, delirium, and insensibility.

(Note.—Ailanthus is especially called for in low forms of typhoid fever, diphtheria, scarlatina, where there is stupor, excoriating discharges from nose, and maybe a livid, purple rash in patches with purple-colored skin showing between the patches. In typhoid states it may be compared with Baptisia; in diphtheria, with Arum-triphyllum.)

HEAD.—The vertigo comes on when rising or moving; accompanied by nausea and cold sweat. (Vertigo especially in closing the eyes, Theridion; vertigo on opening eyes, Thuja).

(NOTE.—In the earlier states of scarlatina, the Ailanthus patient will be drowsy, restless, and anxious, with heat and redness of face (if there be any rash out it will be dark and in patches) and this dizziness on moving. A later stage of the disease will find the patient in a stupor, with muttering delirium, and maybe insensibility. Also with the peculiar rash which may be imperfectly developed.)

The headache of Ailanthus is mostly a dull, stupefying pain; patient is confused and is disinclined for motion or thinking. The head is hot and face red. (Resembling somewhat the Belladonna headache, excepting that Bell. headache is chiefly of a throbbing, beating kind, denoting *active* arterial congestion.)

EYES AND SIGHT.—Smarting and aching; conjunctivitis; purulent discharge, agglutinating lids in morning. Shunning of light. Letters and figures are blurred. Ailanthus has cured in scarlatina cases where there were widely dilated pupils; also suffused and congested eyes; startled look when aroused; pupils dilated and sluggish.

NOSE.—Coryza, thin ichorous discharge without fetor, with rawness in nostrils; nose and upper lip covered with thick, gray-brown scabs. Nostrils congested; also stopped up, causing difficult breathing through them.

(NOTE.—The above symptoms (chiefly clinical) of Ailanthus are very similar to those of Arum-triphyllum. With Arum-t. there is, perhaps, more soreness and rawness of nose and lips, more restlessness and less stupor than with Ailanthus. The Arum-t. patient will pick at the sore nose till it bleeds (also Conium), and the mouth is so sore he won't eat or drink. The peculiar rash of Ailanthus and its stupor chiefly distinguish it.)

FACE.—Face pale; complexion sallow and bilious. Red and hot face. Irregular spots of capillary congestion as in drunkards after a debauch. Eruption miliary, more on face and forehead (than on rest of body).

In cases of suppressed scarlatina rash the face assumed a dark mahogany color. Puffed erysipelatous swelling. Inflamed vesicles on lower lip; ragged ulcer near corner of mouth. (Painful crack in corners of mouth, Cund. and Nat mur.) Tearing pain in face, head, and left upper or lower teeth; worse lying, must walk about; better from pressure and toward morning.

MOUTH, ETC.—Teeth covered with sordes. Tongue dry, parched, and cracked. Also coated with whitish coat, *brown in centre;* or coated white with tip and edges livid.

(NOTE.—The Arum-tri. tongue is apt to be very sore, red, and cracked, not coated.)

THROAT.—Irritability of throat and hawking of mucus. Throat tender and sore on swallowing or inhaling air. Thick, œdematous, and dry, choky feeling in throat. Fauces and tonsils inflamed, with spots of incipient ulceration. In scarlatina the throat is livid, swollen; the tonsils swollen and studded with deep ulcers, oozing a scanty, fetid discharge; externally throat and neck swollen and sensitive.

STOMACH, ETC.—Thirst for cold drinks or brandy. Hunger and distressing sensation of general emptiness during the chill. Water tastes flat. Frequent belching with headache. Vomiting speedily during chill; suddenly on sitting up. Food speedily vomited.

Peculiar feeling, emptiness in stomach.

A feeling of "insecurity" (like Aloes), as if he might have diarrhœa any moment. Burning in both stomach and bowels from excessive smoking.

STOOL.—Thin, watery, forcibly expelled, with burning and griping; passed involuntarily with urine. Urine may be suppressed.

LARYNX AND COUGH.—Aphonia for a day or two before the cough begins. (Hoarseness relieved by coughing, Stann.) Voice almost lost morning on waking. (In morning, Alumen, Carb-v., Caust.; on waking, Nux-m.) Cough is deep and painful; breathing is suppressed; also with headache and congestion of face. Expectoration muco-purulent or bloody.

CHEST, ETC.—Excessive soreness and tenderness of lungs. (Arn.) (Chest so sensitive cannot bear percussion or even auscultation, China.) Tired feeling in lungs, breathing is almost too much exertion. (Weak feeling on talking, Sulph.; talking causes such weakness must desist, Calc.) Dull pain and contracted feeling in region of base of heart and through centre of left lung. Rapid, small, or weak irregular pulse. Pressure of clothes on chest is uncomfortable. (Also Benz-ac., *Lach.*)

LIMBS.—Numbness and tingling in arms and fingers (Acon., Kalm., Rhus) on waking. Large water blisters on end of thumb and sides of finger nails. Left leg feels numb, with tingling and pricking pain in foot and toes. Pain in left foot on walking.

SLEEP.—Disturbed and unrefreshing. Talks, moans, sweats during sleep. Sleepy after a glass of wine. Sleeps best on right side; worse on waking.

FEVER, CHILL, ETC.—Chill preceded by a miliary eruption, worse on face and forehead; during chill intolerable pain in back and hungry, empty feeling. (Chill with hunger, Nux-v., Phos.) After the chill flushes of heat, soreness of lungs, and pain in head.

SKIN.—Skin dry but not hot. (Bell. skin is *hot* even to the touch.) Eruption of miliary rash in patches of a dark, almost livid color; the skin shown between these patches is purplish. The eruption disappears on pressure, but *returns slowly afterward*. (With Bell. the eruption returns quickly, almost at once; it is also finer than that of Ailanthus.)

The Ailanthus eruption is more profuse on chest, face, and (especially) on forehead than on rest of body. It is slow and tardy, often incomplete in appearing, and is more of a livid color than the scarlet color of the usual scarlatina rash. Ailanthus has caused an eruption looking almost exactly like that of measles, but was not accompanied by catarrhal symptoms. It has also produced a sore on the prepuce which looked very much like an incipient chancre; it dried up on ceasing the drug.

PECULIARITIES.—Sensation as of an electric current from head to extremities. Feeling of fullness everywhere. The extreme soreness in lungs. The peculiar rough, livid rash; slowly returning after the pressure. The stupor and muttering delirium.

Ailanthus is to be compared with Amm-c., Arn., Arum-tr., Aloes, Bapt., Bry., Gels., Hyos., Lach., Nitr-ac., Nux-v., Oleum-jecoris, Phyt., Rhus, Stram., Zinc, etc. Nux vomica is its most general antidote.

### ALETRIS FORMOSA.

This drug is useful in weak, debilitated persons, whether caused by defective nutrition or from protracted illness (Psorin). There is weak digestion; least food causes distress (like Carbo-v.); flatulent colic, constipation, etc.

During an attempt at stool there is terrible pain, as if a passage were being forced. (Grasps the seat tightly while at stool, perspiration stands out on patient, who despairs of having a stool, Alumina.)

In women, with these symptoms, we find menses too early and profuse, often with labor-like pains, general weariness of mind and body. Obstinate vomiting during pregnancy. Menorrhagia with symptoms of uterine and ovarian congestion. Prolapsus uteri from atony. A great tendency to abortion in debilitated women (also Carbo-v., Ferr., Secale, Sepia, Sulph.), with weight, pressure in uterine region, or with a tendency to prolapsus uteri (also Podo.).

In its application to cases of abortion Aletris is especially to be compared with:

CAULOPHYLLUM.—Habitual abortion from uterine atony; when threatened there are spasmodic bearing-down pains; pains especially severe in back and loins (like Kali-c.); tremulous weakness; with all these pains the uterine contractions seem feeble. Like the labor pains of this drug, those felt during threatened abortion are tormenting, irregular, and for most part useless.

CIMICIFUGA is especially called for in cases of abortion in women of rheumatic tendency (Caulo., also, but less prominently). In these cases there are cold chills and pricking pains in mammæ. There may also be convulsions with the labor pains. Abortion following fright (also *Acon.*, Gels., *Op.*).

HELONIAS.—The symptoms indicating this drug are very similar to those of Aletris. Both have the weakness, the prolapsus uteri, the weight and pressure in abdomen, etc. The mental condition of Helonias is marked and distinguishes it. The patient is very *melancholy* and depressed; is irritable, cannot bear to be contradicted; she is always better when doing something and when her mind is occupied. This drug is especially suited to women who are enervated from indolence and luxury. Aletris is called for in those who are weak from long sickness or from defective nutrition.

PETROLEUM.—Prolapsus uteri in patients reduced by chronic diarrhœa, occurring during the day.

SABINA is especially called for in women who abort habitually about the third month (others are Apis, Plumb., *Secale*, *Thuja*). The pains go from back through to pubis; also pains in legs. There is apt to be a profuse discharge, bright red and partly clotted.

SECALE.—Also in abortion at third month, especially in thin, feeble women, with passive hemorrhage of dark blood. The pains are feeble and irregular; parts seem to be relaxed, yet no progress is made.

VIBURNUM.—Threatened abortion in cases where the pains begin in back and go around into uterus, where it is a very severe cramp.

Besides the remedies here noticed, the precursory symptoms of abortion are chiefly covered by such remedies as Arn., *Bell.*, Bry., Cannab-s., *Cham.*, China, Cina, Cocc., Croc., Hydras., *Hyos.*, Ipec., Kali-c., Lyc., Nux-v., Plat., Plumb., Puls., Rhus, Ruta, Sepia, Silicea, Sulphur, Zinc, etc.

### ALLIUM CEPA.

MENTAL.—Apathy. Melancholy with catarrh. Restless anxiety (causing restless changing of position from the pains, like Arsenic). Becomes almost distracted from pain in suppuration of fingers.

HEAD.—Confusion of head and headaches *with coryza or catarrh.* (The catarrh may be slight and the headache will be severe.) Pains in temples, worse on moving or winking eyelids. (Pain in temples on moving eyelids is, also, Badiaga, China, and Sulphur.)

Dull headaches, with coryza, worse in evening, in warm room, better out-doors. Headaches ceasing during the menses. (Headache ceasing on appearance of menses, *Verat.;* ceases at the onset of menses, but returns during the flow, Alum.)

Numbness in bones of skull, as if asleep. A prickling sweat on the bald vertex after each meal. (Sweat on forehead while eating, Carbo-v., Nitr-ac., Nux-v.; on scalp, Nux-v., Petr.) With the headache a sensation as if whole head were wrapped up in warm water.

EYES, ETC.—Excessive lachrymation, with redness of the eyeballs, frequent sneezing. Burning, biting, and smarting of eyes, as from smoke. (Crocus has objects appear as if viewed through smoke.) Lachrymation worse in evening in warm room.

EARS.—Pain in ear from deep in the head. Humming and roaring in ears, sounds seem to come from a long distance (also Cham., Ether, Sol-n.).

NOSE.—A most prominent use of this drug is its place in the treatment of nasal catarrh. The discharge is watery and acrid from the nose but bland from the eyes. A watery discharge dropping freely from the nose is very frequently stopped by this remedy. Violent catarrhs coming on after a spell of rainy weather, with northeast winds; the eyes water, much sneezing and dropping of clear water. This remedy has been also found useful in "hay-fevers" or annual August colds, where patient is very sensitive to the odor of flowers, etc. The coryza is worse evenings and in-doors and better out in open air. (This is very similar to the coryza of Nux-v. We have fluent out-doors, stopped in-doors, Iod., Kali-c., Sulph. All-cepa excoriates the upper lip. Mercurius, the alæ and calumnæ of nose; while Arum-tr. excoriates both nostrils and upper lip, the *left* nostril the most.)

FACE.—A peculiar pain in face, described as "thread-like,"

is found with this drug. Paralysis of left side of face (may be also in limbs of same side), with copious secretion of urine.

A toothache which is worse when the catarrh ceases and is better as catarrh is freer.

THROAT, ETC.—Much dryness of mouth. Also an aching like a large lump on the root of tongue, roof of mouth, extending toward the ear. With its influenza, we find soreness and dryness of the throat. Symptoms better in cold air. Among its sensations are numbness and coldness felt in throat (this cold feeling is prominent with Cistus). A tearing pain in throat on coughing (also Cistus and Phosp.), which pains so much the patient grasps the larynx to protect it.

A feeling as of food stuck behind the breast bone and would not go down. Tickling in larynx is quickly relieved by eating a piece of apple.

APPETITE, ETC.—Bad effects from eating spoiled fish (like Carb-v. and Puls.). Hering reported cases (of yellow fever and of pneumonia) where the patients craved raw onions.

ABDOMEN.—Again the "thread-like" pain is noted; cutting like a thread in abdomen. The colicky pains are worse sitting and better when moving about and also after passage of flatus.

ANUS, ETC.—In cases of constipation after the abuse of Quinine, think of this drug. (Chronic weak digestion from abuse of Quinia is Verat-alb.) In anus and the protruding hemorrhoids there is felt a cold creeping, like a worm, worse on left side. (Guernsey gives, crawling sensation, Ant-c., Chin., Kali-c.)

URINE.—Increased secretion, with coryza. Spasmodic strangury after getting feet wet. (Retention of urine from cold is chiefly met with under Aconite; a pain in urethra from getting feet wet is Calc.) Bladder very sensitive. Child cannot bear to have hand placed there, makes it scream.

MALE SEXUAL ORGANS.—Pains in bladder and prostate after coition; a weakness in hips interferes with coition. Strangury of a spasmodic nature after getting feet and bowels cold.

LARYNX, ETC.—Catarrhal laryngitis; a hoarse cough which seems to tear the larynx; the patient grasps it with hand; he crouches from the suffering. There is a constant inclination to hack to relieve the tickling in larynx. The cough is worse in evening and in cold air. He must take a long breath and then he is sure to sneeze.

BACK.—Pain in small of back if bowels did not move. (Backache with constipation is also Kali-bi., Lach.) Intense cervical pains with influenza. Rigidity of spinal column in periodic attacks, with pain through chest.

EXTREMITIES.—Pains in the joints, especially a laming pain. Whitlow or panaritium on fingers, pains very severe and shoot up the arm. Panaritia in childbed. Sore and raw places on the heels from friction. (Compare here with Am-m., Ant-cr., Caus., Graph., Led., Mang., Natr-c., etc. For rheumatism in the heels, when patient cannot bear any weight on the heel, Mang. is often indicated. Plethoric women with arthritic pains often require Sabina.) Neuralgia in stump of an amputated limb (also Am-m.), with violent burning, stinging pains.

GENERALITIES.—The pains of Allium cepa come on after exposure to cold wind and damp weather. It has ailments from getting feet wet (like Calc., Puls., Sep., Sil., etc.). Most symptoms are better out-doors (except cold air aggravates the cough); motion ameliorates many symptoms. Its symptoms occur in spring, in August, or in autumn. Its colds go from left side to the right. All joints ache. Injuries do not heal.

### ALLIUM SATIVA.

MENTAL.—The patient fears he will not be able to take any medicine; fears he will not recover; fears being poisoned. This is evidently a hypochondriacal condition.

HEAD.—We find heaviness and dizziness ceasing during menstruation and returning afterward.

EYES.—Patient has catarrhal ophthalmia, which is brought on when he tries to read at night; accompanied with agglutination of the eyelids.

MOUTH.—Sensation of a hair on tongue during night and renewed in morning on waking. Copious flow of sweetish saliva after meals. Mouth symptoms worse from reading.

(NOTE.—Sensation as of a hair on tongue is Natr-m.; on back part, not relieved by eating or drinking, is Kali-bi.; sensation as of hair on tip of tongue, followed by numbness of mouth, Nat-ph.; sensation of hair on fore part extending to trachea, Silicea.)

STOMACH, ETC.—Voracious appetite and complaints from overeating. The least change in diet causes dyspeptic troubles. Symptoms worse after eating; chest symptoms also. Desire for butter (also Merc.); complaints from using bad water (also Zingiber). Useful in old chronic cases of dyspepsia, especially in old and fleshy patients whose bowels are disturbed and dyspepsia made worse by least change in diet.

ABDOMEN.—Everything seems to drag downward (like Lil-t., etc.). Each step on the pavement causes excruciating pain, as if intestines would be torn apart; better lying. Constant dull aching pain in bowels with constipation. (Pregnancy, etc., said to be injurious to the pregnant or nursing woman.)

LARYNX, ETC.—A chronic catarrh of trachea, etc., without fever, but with difficult breathing and moist cough. Sternum feels compressed.

EXTREMITIES.—Rheumatism of hips (Coloc.). Also an intolerable pain in the common tendon of the iliac and psoas muscles; worse from least (active) motion; cannot cross legs unless he does so by gently lifting the limb; worse night in bed, cannot sleep or change his position. Pains worse from changes of temperature and from moist heat.

GENERALITIES.—Periodic asthma. Dropsy after protracted intermittent fever in marshy country. The chest troubles are worse in open air. Coldness felt in sleep and awakes patient. Pressure relieves pains in abdomen.

