# A Correction

**Published:** 1888-07

**Authors:** 


### A CORRECTION.

Dear Doctor:—Page 219 of your journal, third line, you wrote:

Vertigo, especially on closing the eyes—Theridion.

Vertigo, especially on opening the eyes—Thuja.

I cannot find the source from which you draw that inference. Hering gives it: Vertigo with eyes shut, *ceases* on opening them, etc.

Lippe, in *Repertory:* Vertigo, closing eyes: Apis, Ars., Callad., Grat., Lach., *Thuja, Theridion.*

Allen's *Cyclopœdia*, 164: Feeling of vertigo on closing eyes (Flatarowitch); 169: Vertigo, especially on sitting and closing eyes—it disappears when lying down (Hahnemann); 175: Vertigo as soon as she closes her eyes, everything turns around, vanishing on opening eyes (Wolffe, with a single pellet of M.)

Please tell us who is right.

Yours truly,

S. L.

[Note.—The best authorities, Hering, Lippe, etc., give this symptom as: Vertigo on closing the eyes, *ceasing* on opening them, Thuja.—E. J. L.]

